# MicroRNA-Regulated Pathways in Hematological Malignancies: How to Avoid Cells Playing Out of Tune

**DOI:** 10.3390/ijms141020930

**Published:** 2013-10-18

**Authors:** Alessandro Fatica, Francesco Fazi

**Affiliations:** 1 Department of Biology and Biotechnology “Charles Darwin”, Sapienza University of Rome, Rome 00185, Italy; 2 Department of Medico-Surgical Sciences and Biotechnologies, Sapienza University of Rome, Latina 04100, Italy

**Keywords:** microRNA, leukemia, lymphoma

## Abstract

The coordinated expression and interplay among lineage specific transcription factors and microRNAs contribute to the regulation of gene expression and determination of cell specificity. In hematopoietic stem cells (HSCs), unique combinations of transcription factors largely control growth and maturation of different blood cell lineages through cooperative regulation of specific target genes. MicroRNAs provide an additional level of control beyond transcription factors. By acting as regulators of crucial lineage-specific genetic programs, microRNAs direct early multipotential progenitor cells to adopt a certain cell fate program. Thus, alteration of specific microRNA levels may affect proliferation, differentiation and genetic stability of HSCs, contributing to the onset of myeloproliferative disorders and leukemia. The major aim of this review is to highlight the critical role of microRNA-regulated pathways during the establishment and progression of hematological malignancies, with a particular attention to leukemia, lymphomas and myelodysplastic syndromes. This will give us the opportunity to discuss the potential use of microRNA-based therapeutic approaches in these diseases. MicroRNAs are indeed emerging as relevant tools to improve the efficacy of currently used therapeutic protocols.

## 1. Introduction

Try to imagine that what is happening within our cells as a sort of “symphony”. The music is complex, performed by thousands of players who need to be directed. The recently discovered class of non-coding RNAs called microRNAs (miRNAs) has a crucial part in directing what tune our cells play. miRNAs are small, only 20–22 nt long, but mighty. A single miRNA can control the amount of hundreds of different proteins produced in the cell by regulating their mRNA translation and stability via recognition of complementary target sites in their 3′UTR [1]. The biogenesis of miRNAs, their regulation and mode of action has been extensively covered in different reviews [2,3]. The more than 2000 different microRNAs identified in the human genome might regulate as many as half of our coding genes. MiRNAs regulate some of the most basic processes in cells such as cell proliferation, metabolism, and apoptosis and, above all, they are central in contributing what type of cell a developing cell ultimately becomes [4]. They are like “conductors” of protein orchestras. A tumour can be regarded as tissue in which cells “lose their identity”, become malignant and start to grow in an uncontrolled or inappropriate manner instead of developing into their determined cell type [5].

Deregulation of miRNAs in cancer cells interferes with normal cell development and causes the cells to “play out of tune”. Thus it is not surprising that miRNAs seem to be important players in the initiation and progression of cancer including hematological malignancies [6].

Hematopoiesis is a highly regulated process in which pluripotent hematopoietic stem cells (HSCs) give rise to all the blood lineages: the myeloid lineage, which comprises neutrophils, eosinophils, basophils, monocytes, macrophages, megakaryocytes, platelets and erythrocytes; and the lymphoid lineage, which includes T and B cells. The development of myeloid and lymphoid cells depends on the activation of specific genetic programs that are responsible for the reduction in cell proliferation and the expression of lineage specific genes [7,8]. Among master regulators of these programs are transcription factors [9]. miRNAs provide an additional level of control beyond the transcription factors by fine-tuning differentiation and adjusting the cell response to external stimuli [10]. In particular, they play a crucial role in the regulation of blood cell specification by controlling the precise timing and expression levels of key factors and conferring robustness to regulative networks [11,12].

Aberrant expression of many different miRNAs has been observed in several cancers, including hematological malignancies. Furthermore, about 50% of miRNAs are located at fragile sites and genomic regions in the human genome associated with cancer [13].

In this review, we deal with well-established molecular circuitries between key protein regulators and miRNAs in hematopoietic differentiation and how their deregulation may contribute to hematological malignancies, with a specific focus on leukemia, lymphomas and myelodysplastic syndromes (Table 1). Finally, we also discuss the possible use of miRNA-based therapies as novel clinical approaches in these diseases.

## 2. Oncosuppressor miRNA Pathways

### 2.1. The miR-26 Family

**Figure 1:**
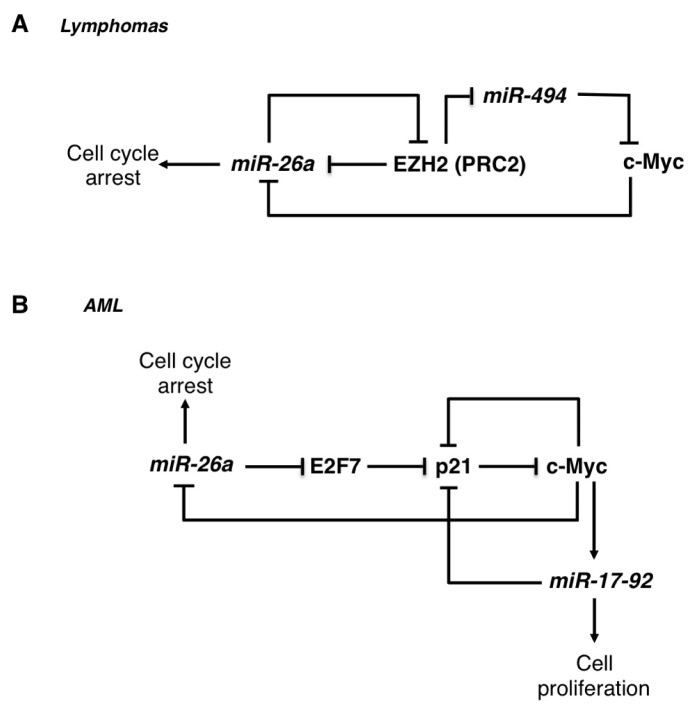
(**A**) Schematic representation of the regulatory network between miR-26a, c-Myc and EZH2 in lymphomas. Repression of miR-26a by c-Myc induced the expression of its target *EZH2*, which support c-Myc oncogenic activity by epigenetically silencing genes involved in cellular differentiation and tumour suppression. EZH2 targets include miR-26a and the c-Myc repressor miR-494 (see text for details); (**B**) Schematic representation of the regulatory network between miR-26a, c-Myc, E2F7 and p21 in AML. Repression of miR-26a by c-Myc induced the expression of its target E2F7, which in turn inhibits the expression of the tumour suppressor p21. p21 suppression is enforced by c-Myc dependent activation of the oncogenic miR-17-92 cluster (see text for details).

The miR-26 family is composed of miR-26a-1, miR-26a-2 and miR-26b. These miRNAs are embedded within introns of three different genes coding for the proteins of the “carboxy-terminal domain RNA polymerase II polypeptide A small phosphatise” (CTDSP) family: *RBSP3*/*CTDSPL* (miR-26a1), *CTDSP2* (miR-26a-2) and *CTDSP1* (miR-26b). The miR-26 family is transcriptionally repressed by the c-Myc oncogene, which is highly up-regulated in many different types of hematological malignancies, including Burkitt lymphoma, B-cell lymphoma, and diffuse large-B-cell lymphoma and different types of leukemia [51,52]. Moreover, in mouse models c-Myc overexpression is sufficient to induce myeloid leukemia and its activation is necessary to develop B-cell lymphoma [53,54]. miR-26 family is often down-regulated in various cancers, and different works have demonstrated that it might act as a tumour suppressor in lymphoma and acute myeloid leukemia [23,24,55,56]. Indeed, ectopic miR-26a expression in different cellular model systems impaired cell cycle progression and attenuated cell proliferation [23,24]. One of the relevant downstream effectors of the c-Myc/miR-26 network is the histone methyltransferase EZH2, a component of the polycomb repressor complex 2 (PRC2) [57]. This complex is often up-regulated and plays a key role in the aberrant transcriptional gene silencing that occurs in different tumours and it was recently shown to participate in the c-Myc dependent miR-26a transcriptional repression [56]. In Burkitt lymphoma, miR-26a can directly regulate the translation of *EZH2* mRNA. Therein, c-Myc can contribute to *EZH2* activation by repressing its negative regulator miR-26a [23]. Furthermore, EZH2 sustains c-Myc expression via inhibition of c-Myc mRNA targeting by miR-494, producing a positive feedback regulatory loop [56] (Figure 1A). A separate mechanism of *EZH2* regulation has been described in AML, in which c-Myc directly activates the transcription of the *EZH2* gene [55]. In AML, miR-26a directly regulates the expression of the E2F7 protein, a transcriptional repressor of the cyclin-dependent kinase inhibitor *p21^CIP1/WAF1^* (also known as *CDKN1A*) [24] (Figure 1B). Moreover, down-regulation of E2F7 levels by miR-26a results in inhibition of c-Myc transcriptional activity, leading to the down-regulation of the c-Myc transcriptional target miR-17-92 cluster [24], whose expression has a well-defined role in contributing to block monocytic differentiation and sustain AML cell proliferation (see below).

Notably, miR-26 host genes encode for proteins that act, similarly to miR-26, as oncosuppressors in AML by controlling the phosphorylated form of pRb [58]. In AML *RBSP3*/*CTDPSL* is negatively regulated by miR-100, a microRNA up-regulated in this tumour, leading to an increase in phosphorylated pRB levels and increased cell proliferation [59].

Conversely, miR-26a was found to promote T-cell acute lymphoblastic leukemia (T-ALL) through the inhibition of the oncosuppressor *PTEN* [25]. This dual activity of miR-26 as oncosuppresor and oncogenic molecule was also described in different solid tumours [60,61]; it thus must be seriously considered in future miRNA-based therapy.

### 2.2. The miR-29 Family

The miR-29 family includes miR-29a, miR-29b-1, miR-29b-2, and miR-29c, encoded in two gene clusters (miR-29b-1/a and miR-29b-2/c). Members of the miR-29 family were found down-regulated in AML with *MLL* rearrangement and deleted in AML with loss of chromosome 7q, which encoded the genes for miR-29b-1 and miR-29a [27,28]. Accordingly with miR-29 expression level in AML, a miR-29 oncosuppressor activity was described by its ectopic expression in AML cell lines and primary AML blasts, where it induced apoptosis and inhibited proliferation, and by inoculation of miR-29b mimics into xenografts, where it decreased tumour growth [27,28]. Moreover, miR-29a was shown to promote myeloid differentiation of human hematopoietic stem/progenitor cells [62]. By contrast, in a different study oncogenic activity of miR-29a was described and it was shown that forced expression of miR-29a in mouse myeloid progenitors cells *in vivo* altered myeloid differentiation and caused AML [63]. This opposing function in leukemogenesis was also observed in B-cell chronic lymphocytic leukemia (B-CLL). Indeed, in aggressive forms of B-CLL miR-29b was found down-regulated by an epigenetic mechanism that involves histone deacetylases (HDACs) [64] and an oncosuppressor activity for miR-29 was suggested through the translational inhibition of the *TCL1* oncogene [65]. By contrast, a different situation was identified in the indolent form of B-CLL, where miR-29a is generally up-regulated compared to normal B-cells. Furthermore, overexpression of miR-29a in mouse mature and progenitor B-cells produced CLL, at least in part, by targeting the tumour-suppressive cell-adhesion molecule peroxidasin homologue (*PXDN*) [66], indicating that miR-29 can predispose cells to a cancerous state and contributes to the pathogenesis of indolent CLL [66]. Thus, these data show that, similarly to other miRNAs, miR-29 may function as a tumour suppressor or oncogene, depending on the cellular context. This may reflect the existence of cell-specific auxiliary factors that may regulate miR-29 function, such as RNA binding proteins or RNA modifying enzymes [67], or off-target effects.

**Figure 2:**
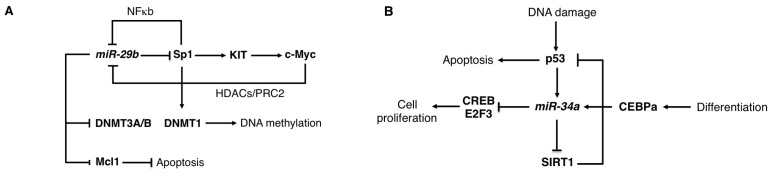
(**A**) Schematic representation of the regulatory networks mediated by miR-29b in hematological malignancies. miR-29b affects DNA methylation by direct and indirect targeting of *DNMT3A*/*B* and *DNMT1*, respectively. In addition, it represses c-Myc activity through translational repression of Sp1 and stimulates apoptosis by inhibiting Mcl1 (see main text for details); (**B**) Schematic representation of the regulatory networks mediated by miR-34a in leukemia. During myeloid differentiation CEBPα induces miR-34a, which post-transcriptionally represses the transcriptional regulators E2F3 and CREB. This results in decreased cell proliferation. In addition, miR-34a is a transcriptional target of p53 and sustains its expression through inhibition of SIRT1 (see main text for details).

The oncosuppressor activity of miR-29 involved, at least in part, regulation of DNA methylation [27]. Aberrant DNA methylation plays a central role in AML by repressing the expression of pro-differentiative genes. miR-29b was initially identified as a repressor of the DNA methyltransferases, *DNMT1*, *DNMT3A* and *DNMT3B* [27]. *DNMT3A* and *DNMT3B* are direct targets of miR-29b, while *DNMT1* is indirectly regulated through the miR-29b target Sp1, a transcription factor that activates *DNMT1* transcription (Figure 2A). An identical function for miR-29s in global DNA methylation was also described in multiple myeloma (MM) [29]. miR-29 levels are regulated by c-Myc and AML with activating mutations of the tyrosine kinase receptor *KIT*, which induces cell proliferation and survival, are characterized by increased c-Myc repression on miR-29b transcription [30]. This leads to increased levels of the miR-29b target *Sp1* that, in turn, stimulates *KIT* transcription in association with NFκB [30]. Furthermore, the Sp1/NFκB complex recruits HDACs on the miR-29b promoter region, which results in a further Sp1 increase. Of note, the *in vivo* disruption of the Sp1/NFκB association in a mouse model for *KIT*-driven leukemia results in a strong inhibition of leukemogenesis and suggests that the targeting of the miR-29b/Sp1/NFκb regulatory loop represents a viable therapeutic option in AML [30].

In mantle cell lymphoma and other Myc-associate lymphomas, miR-29b is repressed by the c-Myc oncoprotein in cooperation with HDACs and PRC2 [68]. In these lymphomas, the PRC2 activity is sustained by c-Myc dependent inhibition of a second oncosuppresor miRNA, miR-26a (see above). The proliferative cycline dependent kinase CDK6 was identified as a relevant target of miR-29b in these B-cell malignancies [26]. Notably, restoration of miR-29b level expression by simultaneous inhibition of HDAC3 and EZH2 inhibited lymphoma growth both *in vitro* and *in vivo* [68], therein expanding the use of miR-29b as a therapeutic option in hematological malignancies.

### 2.3. The miR-34 Family

The miR-34 family consists of three members: miR-34a, miR-34b and miR-34c. miR-34b and miR-34c are produced from a common non-coding transcript. The miR-34 family is a transcriptional target of the oncosupressor p53 and contributes to its downstream effects on proliferation arrest and induction of apoptosis [69,70] (Figure 2B). miR-34 family members are down-regulated in many tumours with inactive p53, including hematological malignancies [71]. miR-34a is also transcriptionally activated by CEBPα (CCAAT/enhancer-binding protein-alpha), a crucial transcription factor for normal granulopoiesis that is frequently disrupted in AML [9]. The E2F3 and CREB (cyclic AMP-responsive element binding protein) transcription factors were identified as relevant miR-34 targets in AML [31,32]. Furthermore, restoration of miR-34a in primary AML cells carrying *CEBPα* mutations stimulated granulocytic differentiation [32]. In chronic lymphocytic leukemia (CLL) low expression of miR-34a was found in chemotherapy-refractory disease associated with deletion of the *p53* gene, which results in decreased DNA damage response and apoptosis resistance [72]. The TCL1 (T-cell leukemia/lymphoma 1) transcription factor and the sirtuin protein SIRT1 (silent information regulator 1), which are overexpressed in most cases of CLL and play a central role in cell death inhibition, were identified as a relevant miR-34 target [33,34]. In CLL, miR-34a restoration was achieved by using the chemical agent nicotinamide, which specifically blocks proliferation and promotes apoptosis of leukemic cells with a functional p53 [34]. Nicotinamide functions as an inhibitor of the sirtuin protein family, which inactivates p53 by deacetylating a critical lysine residue, by a dual mechanism: (i) through an enzymatic inhibition and (ii) through miR-34a induction and *SIRT1* mRNA translational repression [34]. Furthermore, the use of nicotinamide in combination with DNA-damaging chemotherapeutic agents, which activate p53, has been proposed as a novel therapeutic option of CLL with functional p53 [34]. Notably, therapeutic benefit with systemic delivery of miR-34a has been already achieved in different solid tumours mouse model systems [73–75], indicating that miR-34 delivery represents a viable therapeutic option for leukemia.

### 2.4. The miR-146 Family

The miR-146 family includes miR-146a and miR-146b, produced by two different non-coding transcripts. The miR-146a family member plays an important role in different hematological malignancies, myeloproliferative diseases and autoimmunity. miR-146a expression is specifically regulated in different hematopoietic cell types and during inflammation. In myeloid cells, miR-146a transcription is regulated by the master hematopoietic transcription factor PU.1 [76] while in lymphocytes it is controlled by lineage-specific factors, such as c-ETS [77]. During inflammation, the inducible NFκB boosts miR-146a expression while during lymphomagensis its expression is repressed by the oncogene c-Myc [52].

miR-146a down-regulation characterized the 5q-syndrome, a common subtype of myelodysplastic syndromes (MDS) and miR-146a knockout mouse models recapitulate many features of the human syndrome [41,42]. The tumour necrosis factor receptor-associated factor 6 (*TRAF6*) was identified as a relevant miR-146a target and, overexpression of *TRAF6* in mouse was sufficient to phenocopy the miR-146a knockout model. Notably, with aging, miR-146a knockout mice develop myeloid expansion and a predisposition to develop myeloid cancer [41,42]. It was speculated that this was the result of increased NFκB levels and the activation of a pathologic hematopoietic program called inflammatory hematopoiesis. Furthermore, a small percentage of miR-146a knockout mice also developed different types of lymphomas [42]. Altogether these data indicate a tumour-suppressor role for miR-146a in hematological malignancies. However, in breast cancer an oncogenic activity for miR-146a was characterized [78].

miR-146a is also important for regulating the normal development of hematopoietic cells and it plays also an important role in the control of mature cell function in the innate immune system, where it acts as a negative regulator of inflammation [42]. Moreover, miR-146a was found significantly deregulated in autoimmune diseases suggesting an important role for miR-146a also in immunity [79].

### 2.5. miR-223

**Figure 3:**
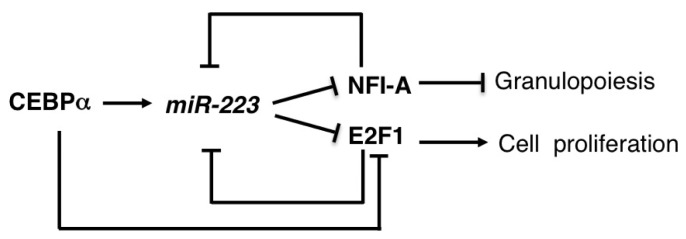
Schematic representation of the regulatory networks mediated by miR-223 in myeloid cells. CEBPα induces miR-223, which post-transcriptionally represses the transcriptional regulators E2F1 and NFI-A during granulopoiesis. This feed-forward loop is connected with feedback loops in which E2F1 and NFI-A inhibits the transcription of miR-223 in undifferentiated myeloid precursors. In addition, CEBPα itself inhibits transcription of the cell-cycle regulator E2F1.

miR-223 expression is mainly linked to the determination of myeloid cell lineage differentiation. miR-223 expression is induced during granulocytic differentiation of human myeloid progenitors and is transcriptionally modulated by the competitive binding of two transcription factors, the Nuclear Factor I (NFI-A) and the CEBPα, to the CAAT elements present on the upstream region to the pre-miR-223 sequence [47]. NFI-A transcriptional activity maintains miR-223 at low levels, while its replacement by CEBPα results in miR-223 up-regulation and granulocytic differentiation. NFI-A is also a target for miR-223 translational repression, thus establishing a feedback regulatory circuitry that is crucial in the control of granulopoiesis (Figure 3) [47]. In agreement with the central role of NFI-A suppression in the induction of human granulopoietic lineage differentiation, the contribution of miR-223 to the *NFI-A* transcriptional regulation was also recently described [80]. During granulocytic differentiation miR-223 localizes in the nucleus and, interacting with complementary binding sites in the *NFI-A* promoter, contributes, together with the Polycomb/RNAi complex, to the induction of heterochromatin formation and to *NFI-A* transcriptional silencing [81]. miR-223 is expressed at low levels in pluripotent hematopoietic stem cells (HSCs) and common myeloid progenitors and its levels increase with neutrophilic differentiation. Of note, miR-223 is down-regulated in different subtypes of acute myeloid leukemia (AML). In particular, low levels of miR-223 expression are detected in primary leukemia blasts carrying the t(8;21) generating AML1/ETO, the most common acute myeloid leukemia-associated fusion protein [82]. In these samples, by recruiting chromatin-remodeling enzymes at an AML1-binding site on the upstream region of pre-miR-223 gene, AML1/ETO induces heterochromatic silencing of miR-223 and block of differentiation. Of note, enforced expression of miR-223 in primary AML blasts enhances granulocyte differentiation, whereas miR-223 knockdown reduces the efficient granulocytic differentiation response to Retinoic Acid (RA) of myeloid progenitors cells [47,81,82].

Moreover, it has been recently shown that, during granulocytic differentiation, miR-223, by targeting the cell-cycle master regulator E2F1, blocks the cell-cycle progression of myeloid cells. In a negative feedback loop E2F1 binds to the miR-223 promoter in AML blast cells and inhibits miR-223 transcription, suggesting that E2F1 is a transcriptional repressor of the miR-223 gene in AML cells (Figure 3) [48].

Strikingly, in T-ALL with aberrant activation of the oncogene *TAL1*, miR-223 is transcriptionally up-regulated by TAL1 itself and contributes to leukemogenesis through the translational repression of the tumour suppressor FBXW7 [49], once again indicating that the effect of a specific miRNA strongly depends on the cellular context.

### 2.6. miR-328

miR-328 is involved in the differentiation and survival of chronic myeloid leukemia cells (CML) by a dual functional activity. Recently, Ering *et al*. [50] demonstrated that miR-328, in addition to the typical base pairing recognition of target mRNA, can also interfere with the mRNA-regulatory function of RNA-binding proteins (decoy activity) [50]. In CML blasts the BCR/ABL fusion protein activates the RNA binding protein hnRNP E2 that, by binding the 5′UTR of the *CEBPα* mRNA, inhibits its translation and myeloid differentiation. During myeloid differentiation miR-328 specifically competes with *CEBPα* mRNA for binding to hnRNP E2 and the interaction between miR-328 and hnRNP E2 leads to the release of *CEBPα* mRNA from hnRNP E2-dependent translational inhibition, resulting in induction of the CEBPα protein production and myeloid differentiation. At the same time CEBPα stimulates miR-328 transcription, thus forming a positive feedback loop that fine-tunes the regulation of myeloid differentiation. In addition to this mechanism miR-328 can also induce the common translational inhibition of the oncogene PIM1, inhibiting cell cycle progression and survival of leukemic BCR/ABL positive blast cells [50].

## 3. Oncogenic miRNA Pathways

### 3.1. The miR-17-92 Family

**Figure 4:**
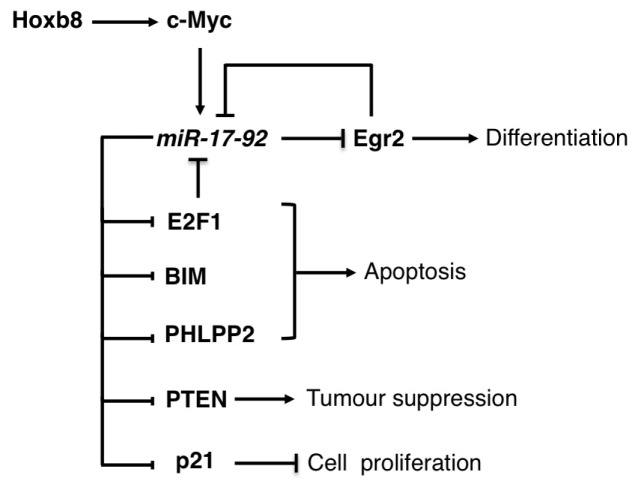
Schematic representation of the regulatory networks mediated by miR-17-92 cluster in hematological malignancies. miR-17-92 exerts its oncogenic activity by affecting multiple pathways trough the inhibition of the apoptotic proteins E2F1, BIM and PHLPP2; the tumour suppressors PTEN and p21 and the pro-differentiative factor Egr2 (see main text for details).

The oncogenic miR-17-92 family is composed of 3 related polycistronic miRNA gene clusters: miR-17-92 cluster and its paralogs miR-106b-25 and miR-106a-363 clusters [83]. The miR-17-92 cluster, which consists of six miRNAs embedded in a single primary transcript, is amplified in both solid tumours and hematological malignancies [83] and is transcriptionally activated by the oncogene c-Myc and by the E2F transcription factors [84,85]. Furthermore, *E2F1*, *E2F2*, and *E2F3* mRNAs are direct targets of miR-17 and miR-20, establishing an auto-regulatory loop that balances E2F expression in proliferating cells and inhibits E2F1-dependent apoptosis [84–86] (Figure 4). The first evidence of a central role for the miR-17-92 family in hematological malignancies was the discovery that a good percentage of large B-cell lymphomas were characterized by genomic amplification of the miR-17-92 locus [14]. Following this observation, it was shown that in mouse models of B-cell lymphomas the ectopic expression of miR-17-92 cooperates with the oncogenic activity of c-Myc [15,16] and that the overexpression of this cluster in mouse lymphocytes is sufficient to induce a lymphoproliferative disorder of the B and T cell compartments [17]. However, knockout mouse models have demonstrated that the miR-17-92 cluster is essential for B-cell development [87]. Notably, the miR-19a and miR-19-b-1 cluster components are sufficient to mediate the oncogenic activity [16]. Moreover, the PTEN tumour suppressor and the pro-apoptotic BIM proteins (also known as BCL2-like 11) were identified as relevant targets for miR-19 activity in B-cell lymphoma [16]. Similar results were obtained in a mouse model for T-cell acute lymphoblastic leukemia (T-ALL) [18]. In mantle cell lymphoma (MCL), the protein phosphatase PHLPP2, an important negative regulator of the PI3K/AKT pathway, was identified as an additional important target of miR-17-92 miRNAs. As a result of PHLPP2 down-regulation, the overexpression of miR-17-92 activated the PI3K/AKT pathway and inhibited chemotherapy-induced apoptosis of MCL cells [88].

The miR-17-92 cluster was also found overexpressed in AML with *MLL* rearrangements and its expression was suggested to contribute to the self-renewal capacity of leukemic cells [19,89,90]. In addition, inhibition of the miR-17-92 expression cluster has been associated with monocyte/macrophage differentiation of both normal progenitors and AML cells [19,20]. A regulatory loop between the pro-differentiative transcription factor Egr2 and the miR-17-92 cluster has been recently described in AML, in which Egr2 is both a repressor and target of miR-17-92 components [19]. During monocytyc differentiation Egr2 is activated by PU.1 and binds to the miR-17-92 cluster promoter leading to transcriptional repression and, eventually, up-regulation of miR-17-92 targets, including Egr2. By contrast, increased miR-17-92 levels results in low expression of Egr2 and other relevant targets and might contribute to the block of differentiation that characterizes AML cells [19]. The oncogenic *HOX* gene, *HOXB8*, whose increased expression contributes to development of AML, was recently identified as positive regulator of c-Myc and consequently of the miR-17-92 cluster [21]. Moreover, the pro-apoptotic BIM protein was identified as a relevant miR-17-92 target. Therein, it was suggested that one of the mechanisms by which HOXB8 sustains AML cell viability is through the indirect down-regulation of BIM [21].

The two paralog clusters, miR-106b-25 and miR-106a-363, share the ability to promote tumourigenesis with miR-17-92. Moreover, miR-106b and miR-93 regulate E2F1 expression, establishing a negative feedback loop analogous to that observed for miR-17 and miR-20. The cyclin-dependent kinase inhibitor *p21*, a potent negative regulator of the G1-S checkpoint, has been identified as an additional functional crucial target of the miR-17-92 family [22,84,87].

Systemic delivery of antisense oligonucleotides against miR-17 has been successfully utilized to abolish tumour growth in a neuroblastoma mouse model system [91], indicating that inhibition of oncogenic miRNA activity might represent a viable therapeutic treatment in cancer.

### 3.2. The miR-125 Family

The miR-125 family includes miR-125a, miR-125b-1 and miR-125b-2, which are produced by three independent clusters containing other miRNAs [92]. The miR-125b members have a prevalent role in hematopoietic cells and were found up-regulated in different types of myeloid and lymphoid malignancies, and MDS. In particular, miR-125 overexpression was identified in different AML subtypes characterized by specific chromosomal abnormalities, including *AML1/ETO*, *PML/RARα* and *FLT3* translocations, and in Down Syndrome-associated acute megakaryoblastic leukemia (AMKL).

Similarly, miR-125 was found up-regulated also in ALL with insertion or translocation of miR-125b into the immunoglobulin heavy-chain (*IGH*) locus, and in ALL with *TEL-AML1* translocation, the most common childhood ALL [92]. The up-regulation of miR-125b in different types of hematological malignancies suggests a general oncogenic role for this miRNA. Indeed, overexpression of miR-125b in hematopoietic progenitors cells and myeloid cell lines increased proliferation, blocked differentiation and inhibited apoptosis [35,36]. The crucial hematopoietic transcription factor CBFB, the tumour suppressor ABTB1 and the pro-apoptotic protein BAK1 (Bcl-2 antagonist killer 1) were identified as relevant miR-125b targets in leukemia [35,36]. Furthermore, overexpression of miR-125b in mice is sufficient to expand the myeloid and lymphoid progenitors and induces leukemia [36–38].

The protein tyrosine phosphatases PTPN18 and PTPN7, and serine/threonine phosphatases PPP1CA and PPP2CA were identified as central targets for oncogenic miR-125b function *in vivo* [38].

By increasing global tyrosine phosphorylation, up-regulation of miR-125 leads to cytokine hypersensitivity of bone marrow hematopoietic progenitors [39], which results in a pre-leukemic state.

miR-125 also represses the pluripotency factor LIN28 [38,40], and LIN28 knockdown in the hematopoietic system mirrored the overexpression of miR-125b, leading to an increase of myeloid cells [38]. miR-125b is also required for B and T-cell development, and the function of macrophages. Thus, it is also involved in inflammation and innate immunity.

Despite the large number of miR-125 functional studies, little is known about its expression regulation. In malignant myeloid progenitors, miR-125b transcription is activated by the homeobox transcription factor CBX2, which is aberrantly expressed in leukemia [93] while in macrophages, the miR-125b primary transcript is transcriptionally controlled by NFκB and the AKT pathway [94].

In conclusion, these data indicate that the oncogenic miR-125b is a suitable therapeutic target in leukemia.

### 3.3. The miR-155

To date, miR-155 is one of the most relevant and well-characterized miRNAs of the immune system and is emerging as critical element for the fine-tuning of the inflammatory response. Analysis of the genomic organization of the miR-155 locus showed that the mature sequence of this miRNA is embedded in the third exon of a host gene primary transcript (miR-155HG), also referred to as B-cell integration cluster (*BIC*). During the inflammatory response, in both myeloid and lymphoid cells, the regulation of BIC/miR-155 expression is under the control of different transcription factors, such as NFκB and AP-1, that induce *BIC* transcription, or STAT3 and AKT, that appear to down-regulate *BIC* transcriptional activity [94–96]. Also the post-transcriptional regulation of miR-155 biogenesis has emerged to be very relevant for the modulation of BIC/miR-155 expression levels during the inflammatory response [43,97].

The deregulation of miR-155 expression contributes to leukemia and lymphoma development by affecting proliferation and survival of leukemia cells [43,98]. In mice models, the deregulation of miR-155 expression in B cell indeed causes acute lymphoblastic leukemia and high-grade lymphomas [43,99] whereas in myeloid cells it is associated with myeloproliferative disorders [100]. More recently, analysis of miR-155 expression revealed that a high level of miR-155 is associated with a lower complete response (CR) and a worse survival in patients with cytogenetically normal acute myeloid leukemia (CN-AML) [46]. In addition, high levels of miR-155 were also detected in newly diagnosed CN-AML carrying the *FLT3*-internal tandem duplications (*FLT3-ITD*) that predict an unfavorable outcome [101]. Thus, the inhibition of miR-155 expression has been recently proposed as a promising therapeutic strategy for the treatment of patients with CN-AML [102]. In this direction, many efforts have been made in the last years to identify the contribution of the miR-155-regulated pathways to the development of myeloid and lymphoid malignancies.

In myeloid cells, it was shown using different methodological approaches, that miR-155 can directly repress several genes such as *BACH1*, *SLA*, *CUTL1*, *CSF1R*, *JARID2*, *CEBPβ*, *PU.1*, *ARNTl*, *HIF1α* and *PICALM* relevant for myeloid lineage differentiation or for the establishment of myeloproliferative disorder [100]. In addition, through both gain and loss of function approaches, Src homology-2 domain-containing inositol 5-phosphatase 1 (*SHIP1*) was also identified as a direct target of miR-155. Of note, specific knockdown of SHIP1 in the hematopoietic system largely recapitulates the myeloproliferative phenotype described in miR-155-expressing mice [45]. More recently, it was evidenced that the transcription factor HOXA9 is able to regulate miR-155 expression and that miR-155 appears to act as a downstream mediator of HOXA9; HOXA9 capacity to induce myeloid colony formation was indeed reduced in miR-155-deficient BM cells [103].

In lymphoid tissues, where the overexpression of miR-155 results in disseminated lymphoma, characterized by a clonal, transplantable pre-B-cell population of neoplastic lymphocytes [104], a specific miR-155-dependent down-regulation of SHIP and CEBPβ proteins was observed in leukemic pre-B lymphocytes; this results in a block of B cell differentiation and might also induce a reactive proliferation of the myeloid lineage [105]. More recently it has been shown that miR-155 suppresses HDAC4 and BCL6 expression during miR-155-dependent leukemogenesis. This inhibition results in the de-repression of some of the known BCL6 targets like inhibitor of differentiation (ID2), interleukin-6 (IL6), c-Myc, Cyclin D1, and MIP1α/CCL3, all of which may block B cell development at an immature B-cell stage of differentiation and induce uncontrolled cell proliferation [44].

Of note, recent results show that the *in vivo* systemic delivery of miR-155 antisense, encapsulated in unique polymer nanoparticles, or LNA-anti-miR-155 slows the growth of pre-B-cell tumours or inhibits tumour growth in a mouse xenograft model of a low-grade lymphoproliferative disorder, respectively [104,106]. All these findings strongly suggest that miR-155 inhibition may represent a promising therapeutic option for leukemia and lymphoma.

### 3.4. The miR-196b

miR-196b is located in a highly evolutionarily conserved region between the *HOXA9* and *HOXA10* genes on chromosome 7p15.2. During hematopoiesis, expression of *HOX* genes is tightly regulated [107]. The Mixed Lineage Leukemia (MLL) factor regulates *HOX* gene expression by binding to their promoters and providing a gene activation mark on the targeted chromatin [108,109]. Interestingly, the *HOXA* cluster genes, and especially *HOXA4*, *HOXA5*, *HOXA9* and *HOXA*10, are also over-expressed in the *MLL*-rearranged leukemia cells [110,111].

During hematopoietic differentiation, miR-196b expression is high in the short-term hematopoietic stem cells (ST-HSC) and decreases as cells become more differentiated. The miR-196b expression follows the expression pattern of the surrounding genes and depends on the MLL factor as well as its dramatic overexpression in leukemia cells by the oncogenic MLL fusion proteins [112]. While miR-196b over-expression increases proliferation and survival, and also partially blocks differentiation of normal bone marrow hematopoietic progenitor cells, the treatment of MLL-AF9 transformed bone marrow cells with miR-196 antagomir significantly compromises their replating potential [112]. More recent findings also support the oncogenetic role of miR-196b in *MLL*-rearranged leukemia suggesting that miR-196b could contribute to the unfavorable prognosis of *MLL*-rearranged leukemia by repressing simultaneously the expression of both oncogenic (HOXA9/MEIS1) and tumour-suppressor (FAS) target genes [113].

Moreover, a recent study also reported that the high expression of miR-196b is not only restricted to *MLL*-rearranged leukemia but can also be found in other types of leukemia with aberrant activation of *HOXA* genes suggesting a potential role for miR-196b in the tumourigenesis of all *HOXA*-activated leukemias [114].

## 4. miRNA Pathways and Drug Response Efficacy

In recent years many efforts have been made to disclose the molecular processes at the basis of the therapeutic drug response to optimize the efficacy of treatment and to improve the clinical outcomes of hematological malignancies. Different miRNAs are described as reliable biomarkers for risk-stratification and management of leukemia [102]. Moreover, miRNAs involved in the anti-neoplastic drug responses are emerging and the modulation of their expression is becoming a possible central tool to improve the efficacy of current therapeutic protocols.

Cytarabine (or Cytosine Arabinoside), also known as Ara-*C*, is one of the most commonly used drugs in the treatment of AML. Ara-*C*-induced drug resistance is associated with down-regulation of miR-181a levels in leukemia cells. On the contrary, enforced miR-181a expression may regulate the death of Ara-*C*-resistant leukemia cells by targeting caspase-dependent cell death through BCL-2 expression and causing subsequent changes in the apoptosis pathway [115]. Moreover, the ectopic expression of miR-32 in AML cells, resulting in a down-regulation of the pro-apoptotic protein BIM, increases the differentiation response to 1,25-Dihydroxyvitamin D3 and cell survival. Interestingly, the inhibition of miR-32 expression is sufficient to elevate BIM expression and sensitize AML cells to Ara-*C*-induced apoptosis [116].

Decitabine (5-aza-2′-deoxycytidine) and 5-azacitidine are cytosine nucleoside analogs that, when incorporated into DNA sequence, form irreversible covalent bonds with DNA methyltransferases (DNMTs) resulting in the hypomethylation of DNA [117]. miR-29b, which negatively modulates the expression of genes encoding the transcriptional activator Sp1 and DNA methyltransferases (DNMT1, DNMT3A and DNMT3B), as described above, is down-regulated in AML. Of note, it was recently reported that low pretreatment levels of miR-29b in leukemia cells might be associated with worse prognosis and inferior response to the hypomethylating agent decitabine in AML patients [118]. The up-regulation of miR-29b levels and the resulting reduction of known miR-29b targets, after the sequential administration of a specific histone deacetylase (HDAC) inhibitor followed by decitabine, caused a stronger anti-leukemic activity of the combined treatment *in vitro* and *in vivo*. These results open up a novel epigenetic-targeting approach for acute myeloid leukemia [119]. Similar results were obtained by using a novel transferrin-conjugated nanoparticle delivery system for synthetic expression of miR-29b (Tf-NP-miR-29b) in AML cells. The miR-29b induction results in a decreased AML cell growth and impairment of colony formation. Moreover, mice engrafted with AML cells and then treated with Tf-NP-miR-29b had significantly longer survival compared with the Tf-NP-scramble-treated group. Of note, the combined treatment of Tf-NP-miR-29 plus decitabine showed an improvement in the survival of the leukemic mice compared with decitabine alone [120]. Moreover the expression of miR-29b in MM targets DNMT3A and DNMT3B and reduces global DNA methylation. *In vitro* transfection of synthetic miR-29b mimics in MM cells promoted apoptosis and cell cycle perturbations and potentiated the growth-inhibitory effects of the DNMT inhibitor 5-azacitidine, suggesting new strategies based on the combination of DNA-demethylating agents and miRNAs in the treatment of MM [121].

Imatinib has revolutionized the clinical treatment of CML by inhibiting the tyrosine kinase activity of the fusion protein BCR-ABL and thereby causing tumor cell death. Emergence of resistance to Imatinib has become a significant clinical problem. In CML, miR-203 was silenced in human BCR-ABL-positive cells through hypermethylation of its promoter. Interestingly, ABL is one of the miR-203 targets and re-expression of miR-203 dramatically reduces the proliferation of tumor cells in an ABL1-dependent manner. [122]. Drugs that inhibit DNA methylation, such as 5-azacytidine and 4-phenylbutyrate, could reverse the hypermethylated state of the miR-203 promoter and restore miR-203 expression with consequent reduction in BCR-ABL levels [122]. This has therapeutic implications, as currently available demethylating epigenetic drugs, such as decitabine, may represent a valid alternative to CML patients who develop resistance to tyrosine kinase Inhibitors [123]. It has been also shown that Imatinib itself induces a demethylation of the miR-203 promoter, resulting in low expression of targeted BCR-ABL and loss of proliferation of leukemic cells. These results highlight demethylation of miR-203 as one of the molecular mechanisms of Imatinib-induced inhibition of BCR-ABL-positive leukemic cells [124].

## 5. Conclusions

In addition to the use of miRNAs as prognostic markers it is very likely that we will see the development of new therapeutic options based on miRNAs in the clinic in the near future. Recent advances in the field demonstrated the feasibility of manipulating miRNA expression levels as a potential therapeutic strategy for cancer [125]. However, it is important to point out that the oncogenic or oncosuppressor activity of specific miRNAs might strictly depend on the cellular context. Therefore, before moving to the clinic it will be critical to fully understand the function of specific miRNAs that constitute therapeutic targets. Hopefully, by controlling the action of these micro-cell conductors in the right time and in the right place we will be able to affect the type of music that cells play and prevent them from playing “out of tune”.

Finally, even if at the present miRNAs are the most studied regulative non-coding RNAs, other classes of non-coding RNAs, such as long non-coding RNAs (lncRNAs), appear to play a significant role in the regulation of gene expression programmes that occurs in higher eukaryotes [126]. The study of these ncRNAs in cancer is still in its infancy, but important new developments are expected in this field. Therefore, a major objective for future studies will be to re-evaluate and re-design established regulative molecular circuitries in light of the contribution of this complex class of non-coding transcripts.

## Figures and Tables

**Table 1:** Oncogenic and oncosuppressor microRNAs in hematological malignancies.

MicroRNA	Malignancies	Function	Targets	References
**miR-17-92**	Lymphomas	OG	*E2Fs*, *PTEN*, *BIM*, *PHLP2*	[14–17]
	T-ALL	OG	*PTEN*, *BIM*	[18]
	AML	OG	*EGR2*, *BIM*, *P21*	[19–22]

**miR-26**	Lymphomas	OS	*EZH2*	[23]
	AML	OS	*E2F7*	[24]
	T-ALL	OG	*PTEN*	[25]

**miR-29**	Lymphomas	OS	*CDK6*	[26]
	AML	OS	*DNMT3A*, *DNMT3B*, *SP1*, *MCL1*	[27–29]
	B-CLL	OS/OG	*HBP1*, *TCL1*, *PXDN*	[26,30]

**miR-34**	AML	OS	*CREB*, *E2F3*	[31,32]
	CLL	OS	*TCL1*, *SIRT1*	[33,34]

**miR-125**	AML and ALL	OG	*CBFB*, *ABTB1*, *BAK1*, *PTPN18*, *PTPN7*, *PP1CA*, *PP2CA*, *LIN28*	[35–40]

**miR-146**	MDS	OS	TRAF6	[41,42]

**miR-155**	Lymphomas and ALL	OG	*SHIP*, *CEBPβ*, *HDAC4*, *BCL6*	[43–45]
	AML	OG	*BACH1*, *SLA*, *CUTL1*, *CSF1R*, *JARID2*, *CEBPβ*, *PU.1*, *ARNT1*, *HIF1A*, *PICALM*	[46]

**miR-223**	AML	OS	*E2F1*, *NFI-A*	[47,48]
	T-ALL	OG	*FBXW7*	[49]

**miR-328**	CML	OS	*PIM1*	[50]

AML, acute myeloid leukemia; ALL, acute lymphoblastic leukemia; B, B-cell; CLL, chronic lymphocytic leukemia; CML, chronic myeloid leukemia; MDS, myelodysplastic syndromes; OG, oncogene; OS, oncosuppressor; T, T-cell.
